# Supplementary material for: Why we need to report more than 'Data were Analyzed by t-tests or ANOVA'
Source: eLife. 2018 Dec 21;7:e36163. doi: 10.7554/eLife.36163 (PMC6326723; doi:10.7554/eLife.36163)
Supplement: Supplementary file 1. [file elife-36163-supp1.docx]

| **Table S1:** Number of articles examined by journal | | | |
| --- | --- | --- | --- |
| **Journal** | **Articles Screened**  (n = 439) | **Original Research Articles**  (n = 328) | **Original Research Articles with ANOVA**  (n = 225) |
| Cellular Physiology and Biochemistry | 103 | 97 | 74 (76%) |
| Acta Physiologica | 14 | 5 | 4 (80%) |
| Journal of Physiology-London | 76 | 36 | 29 (81%) |
| International Journal of Behavioral Nutrition and  Physical Activity | 10 | 10 | 1 (10%) |
| AJP: Lung, Cellular and Molecular Physiology | 22 | 22 | 18 (82%) |
| Journal of General Physiology | 5 | 3 | 2 (67%) |
| AJP: Endocrinology and Metabolism | 6 | 6 | 4 (67%) |
| Frontiers in Physiology | 92 | 59 | 32 (54%) |
| Journal of Cellular Physiology | 36 | 30 | 18 (60%) |
| AJP: Renal Physiology | 24 | 21 | 15 (71%) |
| AJP: Cell Physiology | 10 | 8 | 6 (75%) |
| Journals of Biological Rhythms | 8 | 6 | 4 (67%) |
| AJP: Gastrointestinal and Liver Physiology | 15 | 10 | 7 (70%) |
| Journal of Applied Physiology | 18 | 15 | 11 (73%) |
| Values are n, or n (% of original research articles). Screening was performed to exclude articles that were not full length original research articles (i.e. reviews, editorials, perspectives, commentaries, letters to the editor, short communications, etc.).  Abbreviations: AJP, American Journal of Physiology | | | |
