# Supplementary material for: Why we need to report more than 'Data were Analyzed by t-tests or ANOVA'
Source: eLife. 2018 Dec 21;7:e36163. doi: 10.7554/eLife.36163 (PMC6326723; doi:10.7554/eLife.36163)
Supplement: Supplementary file 2. [file elife-36163-supp2.docx]

**ANOVA & t-test Abstraction Manual**

**Assessing Eligibility**

1. **Excluded:** Enter **yes** if the paper is not original research; leave blank otherwise. If **yes**, enter article type under **Exclude reason** (i.e. review, letter, editorial, systematic review, meta-analysis, etc.). If the article is not excluded, proceed to the ANOVA questions.

**ANOVA Abstraction**

1. **Includes ANOVA:** Did the study include an ANOVA? (enter **Yes** if the study used ANOVA for any analyses or **no** if the study did not include an ANOVA. If **yes**, proceed to remaining ANOVA questions. If **no**, proceed to t-test questions.
2. **Named factors:** Did the study name what factors were included in each analysis (i.e. time and age, obesity and sex, etc.)? Enter **yes** if all factors are named for all ANOVAs, **no** if factors are not named for any ANOVA and **sometimes** if factors are named for some ANOVAs but not others. **Yes** may indicate that factors were named in the text, or the number of factors was specified in the figure or table legends and the reviewer could easily determine the names of each factor by examining the table or figures.
3. **Specify BTW and WIN Ss Factors:** Were between (independent) and within (non-independent) subjects factors specified when describing the analysis? Enter **yes** if specified for all ANOVAs, **no** if not specified for any ANOVA and **sometimes** if specified for some ANOVAs but not others.
4. **RM ANOVA:** Did the study specify that a repeated measures ANOVA was used? Enter **yes** if the authors clearly stated that a repeated measures ANOVA was used or specified that a factor was treated as a within-subjects factor in an ANOVA; otherwise enter **no**.
5. **ANOVA post-hoc specified:** Enter **yes** if the post-hoc test used was named for all ANOVAs with significant results, **no** if the post-hoc test was not specified for any ANOVAs with significant results, and **sometimes** if the name of the post-hoc test was sometimes specified.
6. **Max number of factors in ANOVA:** Find the ANOVA with the largest number of factors. Enter **1** if that ANOVA has one factor, **2** if the ANOVA has 2 factors and **>2** if the ANOVA has more than 2 factors. Enter **not specified** if the number of factors is not specified for any ANOVA in the paper (i.e. “Data were analyzed by ANOVA” or “We used a multi-way ANOVA”).
7. **Needed multiway:** Did the authors use a one way ANOVA when the study design included two or more factors? Enter **yes** if you find obvious evidence of this error or **no** if you do not find obvious evidence of this error. If **yes**, in the **Oneway_Fig_table** column note the variable name or figure/table number. Yes should be used in cases where the authors compare both factors when analyzing or describing their results.
8. **RM ANOVA needed not reported:** Was there evidence that a RM ANOVA was needed (longitudinal or non-independent data) in a situation where the authors used ANOVA without specifying that RM, between-subjects factors or non-independent factors were included? Enter **yes** if there is evidence of this error and no otherwise. If **yes**, enter the name of the figure, table or variable in the next column (**RM_needed_fig_table**).
9. **Complex ANOVA:** Most ANOVAs will be ANOVA with between subjects factors or repeated measures ANONVAs. Enter **yes** if there was evidence that a more complex type of ANOVA was used (i.e. fixed vs. random factors, etc.) and **no** otherwise.
10. **N factor not reported for all:** Enter **not for RM ANOVA** if the number of factors was not reported for RM ANOVA and **no** otherwise.
11. **Report F statistic:** When stating the results of ANOVAs, did the authors report the F-statistic? Enter **yes** if the F-statistic was reported for all ANOVAs, **no** if the F-statistic was not reported for any ANOVA, and **sometimes** if the F-statistic was reported for some ANOVAs but not others.
12. **Report DF:** When stating the results of ANOVAs, did the authors report the degrees of freedom? Enter **yes** if the degrees of freedom were reported for all ANOVAs, **no** if the degrees of freedom were not reported for any ANOVA, and **sometimes** if the degrees of freedom were reported for some ANOVAs but not others.
13. **Report exact p-value:** When stating the results of ANOVAs, did the authors report the exact p-value? Enter **yes** if the exact p-value was reported for all ANOVAs, **no** if the exact p-value was not reported for any ANOVA, and **sometimes** if the exact p-value was reported for some ANOVAs but not others. Presenting a cutoff (i.e. p<0.05, p<0.01) does not count as reporting an exact p-value unless all exact p-values are specified with the exception of p<0.001.

**T-tests**

1. **Includes t-test:** ***Yes*** if any analysis in the paper was performed by a t-test (paired or unpaired); ***No*** if there is no reported use of t-tests or t-tests were only used as a post-hoc test (i.e. following ANOVA). If the answer to this question is no, leave all remaining questions blank. For the remaining questions, consider only cases where t-tests were the primary analysis. Data that were analyzed by ANOVA, followed by t-tests as a post-hoc tests, should not be included.
2. **Specified paired vs. unpaired:** ***Yes*** if the abstractor can determine whether a paired or unpaired t-tests was used for each analysis performed by t-test; ***sometimes*** if this information can be determined for some t-tests but no others, ***no*** if the abstractor cannot determine whether any t-tests were paired or unpaired. Alternate wordings for unpaired t-tests may include t-tests for independent groups, whereas alternate wordings for paired t-tests may include t-tests for non-independent groups. If the authors use the word paired to refer to comparing two groups, rather than, this does not count. The papers included in this sample contained numerous mentions of paired Student’s t-tests; therefore Student’s t-test was not counted as indicating that the analysis was unpaired unless additional information was provided (i.e. unaired Student’s t-test, Student’s t-test for independent samples).
   1. **Includes Students:** For papers that did not otherwise specify whether paired or unpaired t-tests were used by mentioning unpaired or independent samples t-test, enter **yes** if the paper mentioned using Student’s t-test; **no** otherwise.
3. **Specified equal or unequal variances:** ***Yes*** if the abstractor can determine whether each unpaired t-test did vs. did not assume equal variance; ***sometimes*** if this determination can be made for some t-tests but no others; ***no*** otherwise. If the paper did not include any unpaired t-tests; enter no paired t-tests. Student’s t-test indicates that the test assumed equal variance.
4. **Identify data analyzed by t-test or includes 2-group comparison:** ***Yes*** if the abstractor can identify data that were clearly analyzed by t-tests based on information reported in the statistical methods, text or figure legends. If the authors did not clearly report which data were analyzed by t-tests, assume that two sample comparisons were analyzed by t-test. Answer ***yes*** if the paper contains a two group comparison and **no** otherwise. If the answer to this question is no, leave all remaining questions blank.

For the remaining questions, examine data from question 4 that were identified as being analyzed by t-tests.

1. **Reports t-statistic:** Did the authors report t-statistics for all comparisons? Answer ***yes***, ***sometimes*** or ***no*** if t-statistics were reported for all, some or no comparisons, respectively.
2. **Reports DF or exact n:** Did the authors report degrees of freedom or exact sample sizes for all comparisons analyzed by t-tests? Answer ***yes***, ***sometimes*** or ***no*** if this information reported for all, some or no comparisons, respectively.
3. **Reports exact p-values:** Did the authors report exact p-values for all comparisons? Answer ***yes***, ***sometimes*** or ***no*** if exact p-values were reported for all, some or no comparisons, respectively. If all exact p-values are reported and the only range used is p<0.001 or p<0.0001, answer ***yes***. Answer ***no*** if all p-values are listed as p<0.001 or p<0.001 and no exact p-values are reported, or if these are among the smallest of several different ranges that are reported (i.e. p<0.05, p<0.01).
